# Can a deep learning model based on intraoperative time-series monitoring data predict post-hysterectomy quality of recovery?

**DOI:** 10.1186/s13741-021-00178-4

**Published:** 2021-04-06

**Authors:** Xu Zhao, Ke Liao, Wei Wang, Junmei Xu, Lingzhong Meng

**Affiliations:** 1 Department of Anesthesiology, Yale University School of Medicine, 333 Cedar St, New Haven, CT 06520 USA; 2 Department of Anesthesiology, The Second Xiangya Hospital, Central South University, Changsha, Hunan Province China; 3 Ricoh Software Research Center (Beijing) Co., Ltd., Beijing, China; 4 School of Information Science, Japan Advanced Institute of Science and Technology, Nomi, Ishikawa Japan

**Keywords:** Deep learning, Machine learning, Time-series monitoring data, Hysterectomy, Quality of recovery, Prediction

## Abstract

**Background:**

Intraoperative physiological monitoring generates a large quantity of time-series data that might be associated with postoperative outcomes. Using a deep learning model based on intraoperative time-series monitoring data to predict postoperative quality of recovery has not been previously reported.

**Methods:**

Perioperative data from female patients having laparoscopic hysterectomy were prospectively collected. Deep learning, logistic regression, support vector machine, and random forest models were trained using different datasets and evaluated by 5-fold cross-validation. The quality of recovery on postoperative day 1 was assessed using the Quality of Recovery-15 scale. The quality of recovery was dichotomized into satisfactory if the score ≥122 and unsatisfactory if <122. Models’ discrimination was estimated using the area under the receiver operating characteristics curve (AUROC). Models’ calibration was visualized using the calibration plot and appraised by the Brier score. The SHapley Additive exPlanation (SHAP) approach was used to characterize different input features’ contributions.

**Results:**

Data from 699 patients were used for modeling. When using preoperative data only, all four models exhibited poor performance (AUROC ranging from 0.65 to 0.68). The inclusion of the intraoperative intervention and/or monitoring data improved the performance of the deep leaning, logistic regression, and random forest models but not the support vector machine model. The AUROC of the deep learning model based on the intraoperative monitoring data only was 0.77 (95% CI, 0.72–0.81), which was indistinct from that based on the intraoperative intervention data only (AUROC, 0.79; 95% CI, 0.75–0.82) and from that based on the preoperative, intraoperative intervention, and monitoring data combined (AUROC, 0.81; 95% CI, 0.78–0.83). In contrast, when using the intraoperative monitoring data only, the logistic regression model had an AUROC of 0.72 (95% CI, 0.68–0.77), and the random forest model had an AUROC of 0.74 (95% CI, 0.73–0.76). The Brier score of the deep learning model based on the intraoperative monitoring data was 0.177, which was lower than that of other models.

**Conclusions:**

Deep learning based on intraoperative time-series monitoring data can predict post-hysterectomy quality of recovery. The use of intraoperative monitoring data for outcome prediction warrants further investigation.

**Trial registration:**

This trial (Identifier: NCT03641625) was registered at ClinicalTrials.gov by the principal investigator, Lingzhong Meng, on August 22, 2018.

**Supplementary Information:**

The online version contains supplementary material available at 10.1186/s13741-021-00178-4.

## Background

Perioperative care has two fundamental goals. One is to reduce the incidence of complications, and the other is to enhance recovery to the greatest extent possible. Complications and quality of recovery are related but distinct phenomena (Jammer et al., 2015). Complications negatively impact recovery, while the quality of recovery can still vary among patients who, clinically, do not have any or have comparable complications (Bowyer, Jakobsson, Ljungqvist, & Royse, 2014). The question is how to accomplish these goals. One solution is prognostication, i.e., if we are informed of the level of the risk for a given complication or the potential for an unsatisfactory recovery, we can adjust patient care based on the best evidence to minimize undesirable outcomes (Coulter, Locock, Ziebland, & Calabrese, 2014). Therefore, these at-risk patients should receive enhanced care.

To guide intraoperative care, prognostication must happen before surgery or during surgery. Any prognostication based only on preoperative information misses intraoperative information, which could adversely affect prognostication as the quality of intraoperative care is one of the major determinants of postoperative outcomes (Ljungqvist, Scott, & Fearon, 2017). It is theoretically ideal to incorporate intraoperative information during the prognostication of postoperative courses. To do so, practitioners must collect intraoperative information in real time, feed the data into validated models instantaneously, and use the output to guide intraoperative care in a timely manner (Mathis, Kheterpal, & Najarian, 2018).

Intraoperative data can be categorized into two types: one is time-series monitoring data, such as heart rate and blood pressure, and the other is intervention data, such as medications and fluids given to patients. The time-series monitoring data carry temporal and dynamic information, a unique feature distinguishing themselves from non-time-series intervention data. However, there may be an association between intervention and time-series monitoring data because intraoperative interventions may make a footprint in monitoring, for example, the administration of phenylephrine (i.e., an intervention) increases blood pressure and decreases heart rate (i.e., corresponding change in monitoring). We speculate that this footprint may sometimes make the simultaneous use of intervention and monitoring data in a prediction model redundant. Currently, determining how best to use the intraoperative time-series monitoring data during prognostication remains largely unknown.

During conventional modeling (e.g., logistic regression), processed parameters of the time-series monitoring data, such as the maximum, minimum, mean, and median values, are used in modeling. The concern regarding this approach is the loss of temporal and dynamic information embedded in the time-series data. Deep learning models can uniquely learn from the original time-series data, which may be superior to models that can only learn from processed parameters (Fawaz, Forestier, Weber, Idoumghar, & Muller, 2019a).

In this study, we hypothesized that the InceptionTime deep learning model based on the intraoperative time-series monitoring data can predict the quality of recovery after surgery. We based this study on data collected from the intervention guided by Muscular Oxygenation to Decrease the Incidence of PostOperative Nausea and Vomiting (iMODIPONV) trial. As a result, the data were derived from female patients having laparoscopic hysterectomy.

## Methods

This study was based on data collected in the iMODIPONV trial conducted in female patients having laparoscopic hysterectomy (ClinicalTrials.gov Registration: NCT03641625) (Li et al., 2020). This study was conducted according to the Guidelines for Developing and Reporting Machine Learning Predictive Models in Biomedical Research (Luo et al., 2016).

### Patients

Participants were 18–65-year-old females who had no history of smoking and were scheduled for elective laparoscopic hysterectomy. Their American Society of Anesthesiologists (ASA) physical status classifications were I–II. Patients who were scheduled for vaginal or open hysterectomy, urgent or emergent surgery, or a procedure involving bowel resection were excluded. Patients with major systemic comorbidities or who had undergone chemotherapy or radiotherapy within 3 months before surgery were also excluded.

### Data

The modeling used preoperative, intraoperative intervention, and intraoperative monitoring data (Table 1). Preoperative data included patient demographics, ASA classification, anesthesia-relevant history, comorbidities, and laboratory results. Intraoperative intervention data included anesthetic time, medications, inputs, and outputs. The total of these variables for the entire surgery was used in modeling. Intraoperative monitoring data included time-series heart rate, blood pressure, respiratory rate, pulse oxygen saturation, end-tidal carbon dioxide, and body temperature. We additionally included muscular tissue oxygen saturation data as it was monitored in the iMODIPONV trial. All intraoperative monitoring data were recorded every 2 seconds by a research laptop. The recording started approximately 5 min before anesthesia induction and stopped at the end of surgery.

**Table 1:** Type and nature of the data used in modeling

Type of data	Nature of data
Preoperative data	
Demographics (Age, height, body weight, and BMI)	Numerical data
ASA physical status classification	Categorical data
Anesthesia-relevant history (general anesthesia, spinal anesthesia, nerve block or local anesthesia, postoperative nausea and vomiting, and motor sickness)	Categorical data
Comorbidities (psychiatric disease, neurologic disease, hypertension, cardiovascular disease, pulmonary disease, endocrinologic disease, renal insufficiency, and digestive disease)	Categorical data
Laboratory results (hemoglobin, hematocrit, and creatinine)	Numerical data
Intraoperative intervention data^a^	
Anesthetic time	Numerical data
Propofol, remifentanil, and sufentanil^b^	Numerical data
Crystalloid, urine output, and blood loss	Numerical data
Intraoperative monitoring data	
Respiratory rate and end-tidal carbon dioxide	Time-series data, captured from anesthesia machine
Heart rate, systolic blood pressure, diastolic blood pressure, mean arterial pressure, pulse oxygen saturation and body temperature	Time-series data, captured from vital sign monitors
Muscular tissue oxygen saturation	Time-series data, captured from NIRS-based tissue oximeter

*BMI* body mass index, *ASA* American Society of Anesthesiologists, *NIRS* near-infrared spectroscopy, *iMODIPONV trial* the intervention guided by Muscular Oxygenation to Decrease the Incidence of PostOperative Nausea and Vomiting (iMODIPONV) trial

^a^For the intraoperative intervention data, the totals of different variables for the entire surgery were used in modeling

^b^Sufentanil was the standardized opioid for pain control in the iMODIPONV trial

For time-series data, we regarded values that fell outside the 0.5th and 99.5th percentiles as outliers and treated them as missing data. The missing time-series data were filled using values corresponding to the immediately preceding time points. Time-series data varied with respect to recording duration due to variations in surgical time across patients. We scaled all time-series data to the same extent of 1000 time points using standard down-sampling or up-sampling methods (spline interpolation). We chose 1000 time points because our preliminary analyses indicated that models using 1000 time points had non-inferior performance and could be trained faster than otherwise (eTable 1 in Additional file 1).

In the deep learning model, we converted non-time-series data in the form of a single value per feature per patient to time-series data by replicating the value across all time points. In all models, categorical data were converted into binary data using the one-hot encoding method (Potdar, Pardawala, & Pai, 2017). Missing numerical data were filled using mean imputations. All continuous data, including time-series monitoring data, were normalized to a range from 0 to 1. The upper and lower limits used in normalization are presented in eTable 2 in Additional file 1.

### Outcome definition

In this study, we targeted the quality of recovery as an outcome measure, which was assessed using the Quality of Recovery-15 (QoR-15) scale on postoperative day 1 (Stark, Myles, & Burke, 2013). The QoR-15 scale, ranging from 0 to 150, is a validated patient-reported measure of the quality of recovery (Myles et al., 2018). We dichotomized the quality of recovery into satisfactory if the QoR-15 score ≥122 and unsatisfactory if <122. This cutoff value was consistent with a previous study that categorized the quality of recovery as excellent or good if the QoR-15 score ≥122, and moderate or poor if the QoR-15 score <122 (Kleif & Gögenur, 2018). We also referenced the mean and median QoR-15 values of our patient population during the determination of the cutoff value.

### Model development

**Fig. 1:**
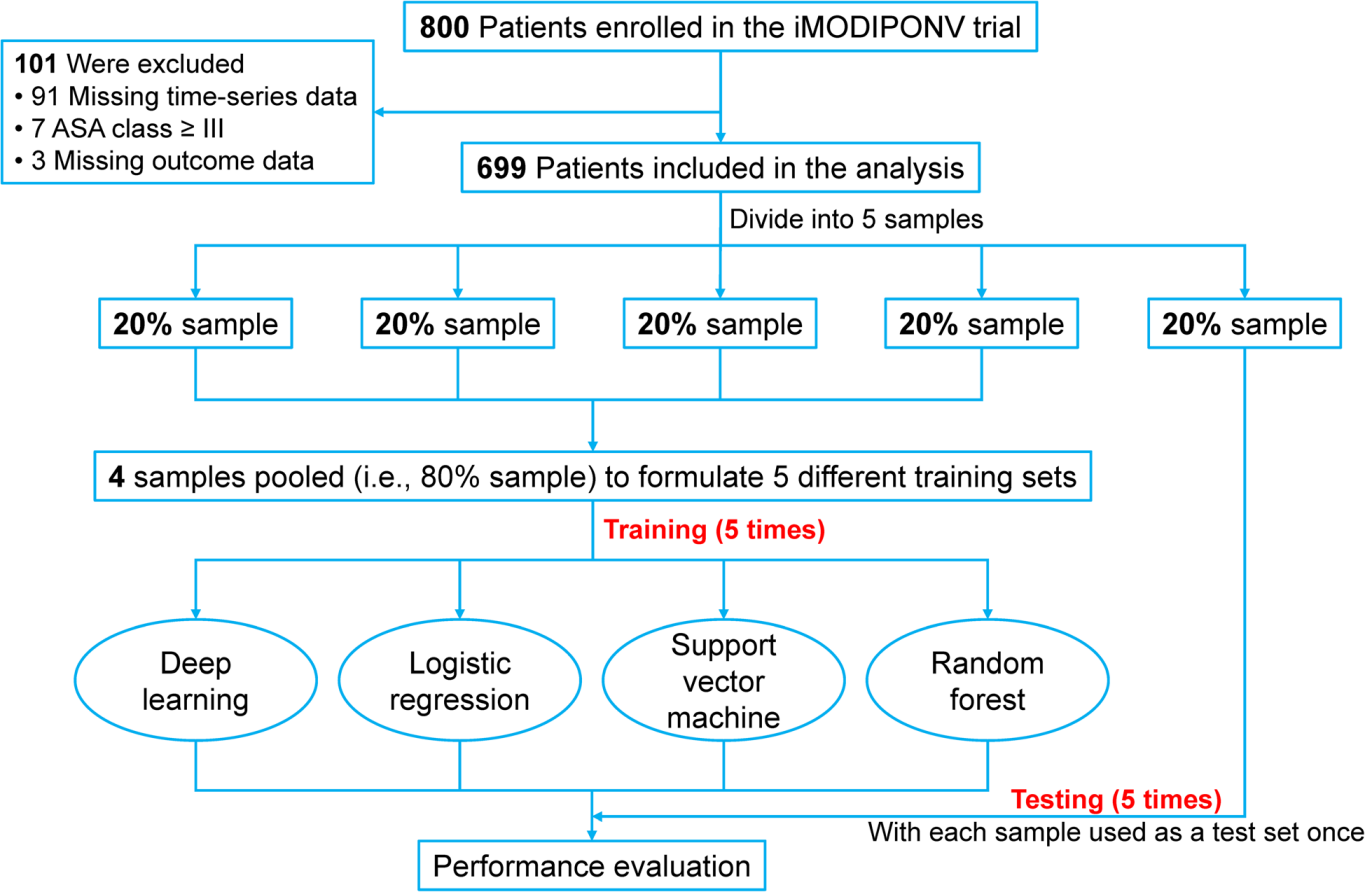
Model development and evaluation. iMODIPONV trial, intervention guided by Muscular Oxygenation to Decrease the Incidence of PostOperative Nausea and Vomiting; ASA American Society of Anesthesiologists

Stratified, 5-fold cross-validation was used to develop training and testing sets (Fig. 1).

### Deep learning model

**Fig. 2:**
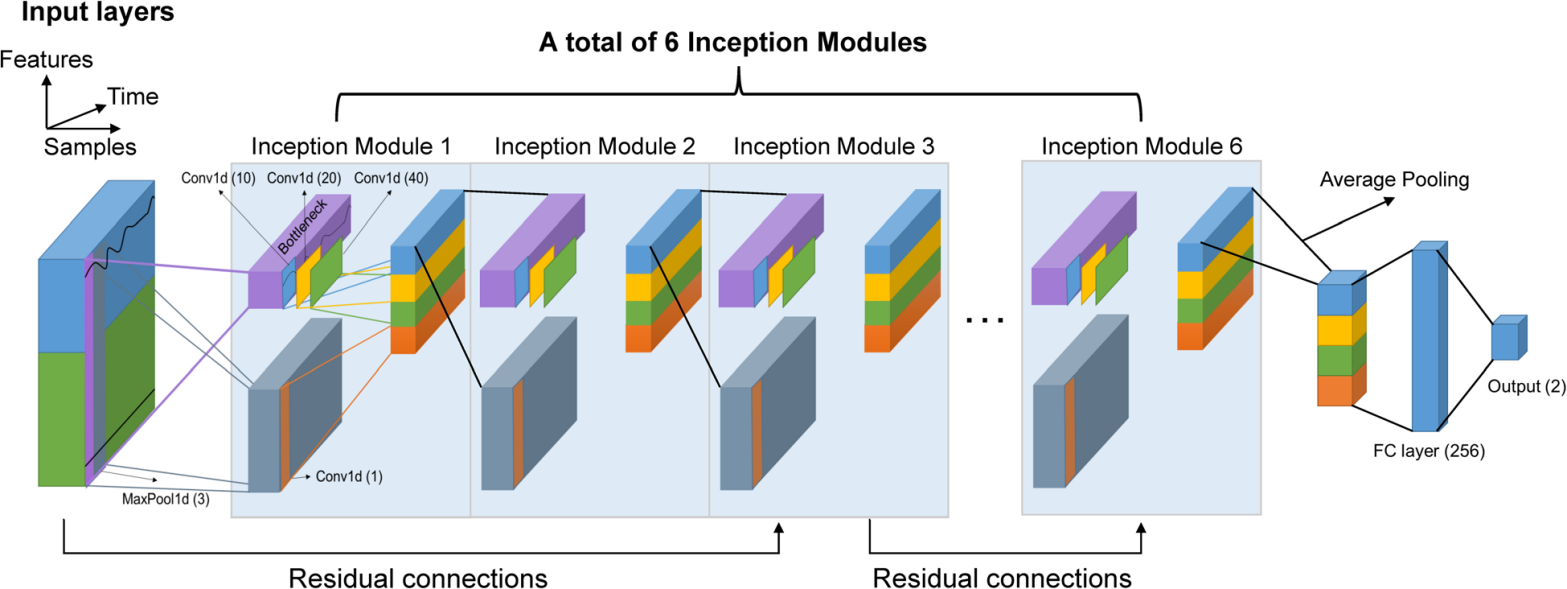
Architecture of the deep learning model. Conv1d one-dimensional convolution, FC fully connected

The architecture of the deep learning model is presented in Fig. 2. The model was based on InceptionTime (Fawaz et al., 2019a, b), which ensembled six sequentially stacked deep convolutional neural network modules (Inception module). In each inception module, the multi-dimension time-series data were transformed into one-dimension data (bottleneck). This process reduced the dimensionality of the time-series data and potentially avoided overfitting small datasets. Three one-dimension filters with lengths of 10, 20, and 40 were applied simultaneously to the output of bottleneck (convolution). A parallel operation was performed to avoid the influence of small perturbations. A window with a length of 3 was slid onto the original multi-dimension time-series data, and the maximum value in this window was computed (MaxPooling). The outputs of each independent parallel convolution and MaxPooling were concatenated to form the output of the current Inception module. The Inception network classifier contained two different residual blocks to mitigate the vanishing gradient. Each residual block was comprised of three Inception modules. Ten percent of patients in the training set were reserved and used as a validation set during the training of the deep learning model. The binary cross-entropy loss with sigmoid layer was used as a loss function. To avoid model overfitting, the training process was stopped when the validation loss began to increase.

### Other models

We compared the deep learning model to three widely used machine learning algorithms, including logistic regression, support vector machine, and random forest. Because these algorithms cannot handle the original time-series data, the maximum, minimum, mean, and standard deviation (SD) values of each time-series data were used in modeling. Default parameters of the scikit-learn (version 0.22.2.post1) were used in the training process of these algorithms.

### Model performance

Accuracy, sensitivity, specificity, F1 score, and area under the receiver operating characteristics curve (AUROC) were used to estimate model discrimination (Alba et al., 2017). Calibration (goodness of fit) was visualized using the calibration plot (Alba et al., 2017). Calibration reflects the extent to which the expected (predicted from the model) and observed outcomes agree. The calibration plot was graphically depicted using the observed outcome frequencies on the ordinate plotted against the expected outcome probabilities on the abscissa. The better the model was calibrated, the closer the points approximated the perfectly calibrated diagonal traveling from the bottom left to the top right in the graph. The overall agreement between the predicted and observed outcomes was quantified using the Brier score (Rufibach, 2010). The Brier score ranges from 0 to 1 and is the mean squared difference between the predicted and observed outcomes. A lower Brier score indicates improved model accuracy.

### Feature importance

For the deep learning model, class activation mapping was used to visualize the contributions of different parts of time-series data to the prediction (Zhou, Khosla, Lapedriza, Oliva, & Torralba, 2016). Class activation mapping provides visual explanation for convolutional neural networks by highlighting the significance of contribution based on local backpropagation. In this study, we used class activation mapping to explore whether any parts of the input appeared peculiar that might confuse the network.

For the logistic regression, support vector machine, and random forest models, the SHapley Additive exPlanation (SHAP) approach was used to appraise the significance of the contribution made by different input features to the prediction (Lundberg & Lee, 2017). The SHAP method is based on the game theory approach that assigns each feature a SHAP value. A larger absolute SHAP value represents a bigger contribution made by the feature to the prediction. We used the fold that had the best prediction performance to evaluate feature importance.

### Software

Model development and evaluation were performed using Python 3.6.9. The deep neural network models were developed using the PyTorch (version 1.0.1), timeseries (version 0.0.6), and fastai2 (version 0.0.19) modules. The logistic regression, support vector machine, and random forest models were developed using the scikit-learn module (version 0.22.2.post1). Performance metrics were calculated using the scikit-learn module (version 0.22.2.post1).

## Results

### Patient characteristics

**Fig. 3:**
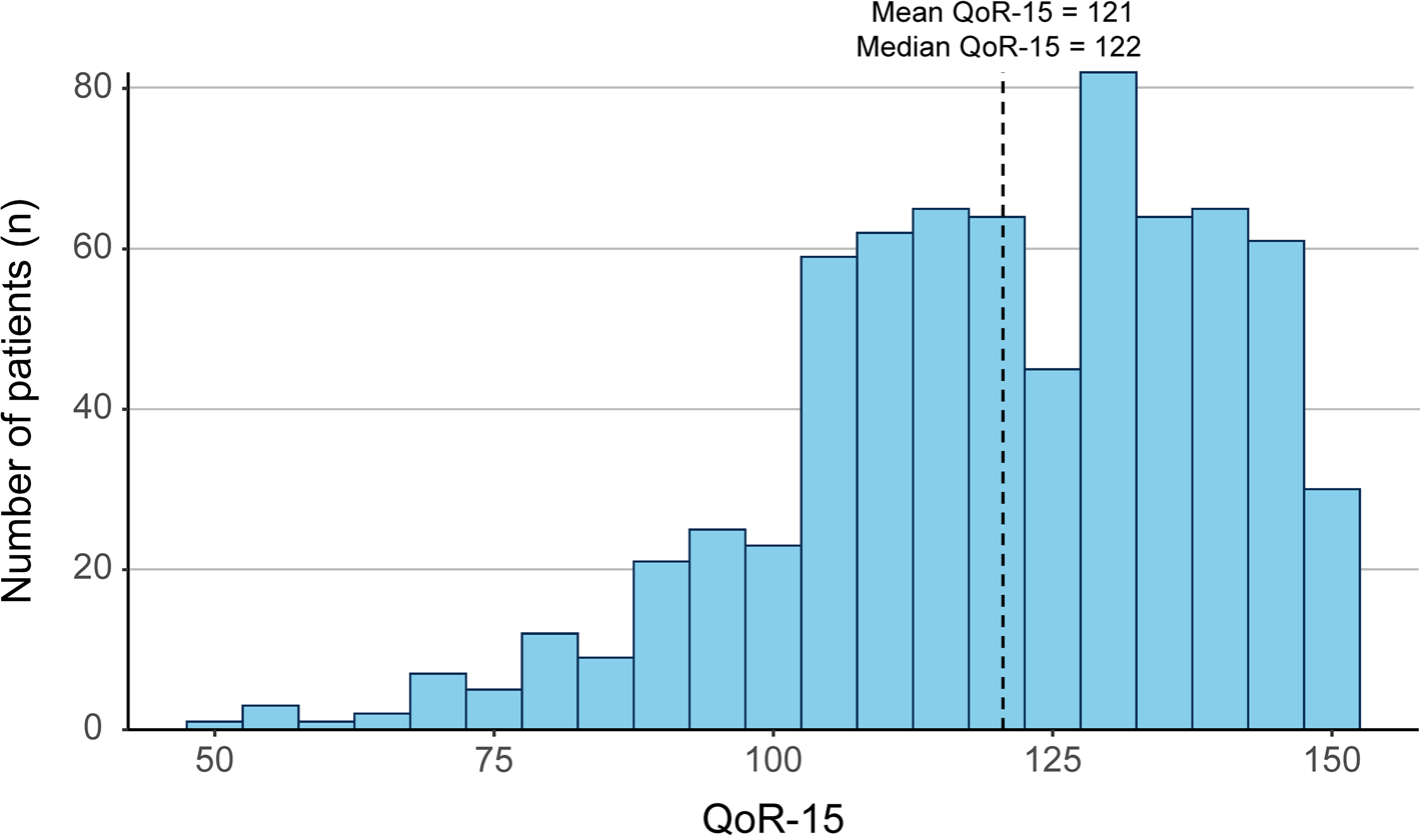
QoR-15 distribution. QoR quality of recovery

The iMODIPONV trial enrolled 800 patients. Of these 800 patients, we excluded 101 patients including 94 patients due to missing monitoring or outcome data and 7 patients due to an ASA classification ≥III (Fig. 1). A total of 699 patients (age, 50±7 years; body mass index, 25±3 kg/m^2^) were included in this study (Table 2). The mean and median QoR-15 scores of our patients were 121 (19, SD) and 122 (109–135, IQR), respectively, and 50.6% (354/699) of our patients had a QoR-15 score ≥122. The distribution of the QoR-15 score is presented in Fig. 3.

**Table 2:** Perioperative data (*n* = 699)

Preoperative data	
Mean age ± SD, year	50 ± 7
Mean body mass index ± SD, kg/m^2^	25 ± 3
ASA physical status, no. (%)	
I	229 (32.8)
II	470 (67.2)
Coexisting medical condition, no. (%)	
Psychiatric disease	3 (0.4)
Neurological disease	15 (2.1)
Hypertension	142 (20.3)
Cardiovascular disease	26 (3.7)
Pulmonary disease	8 (1.1)
Endocrinological disease	69 (9.9)
Renal insufficiency	2 (0.3)
Digestive disease	22 (3.1)
History of anesthesia, no. (%)	
Never	286 (40.9)
General anesthesia	197 (28.2)
Spinal anesthesia	182 (26.0)
Nerve block	2 (0.3)
Local anesthesia	57 (8.2)
History of PONV, no. (%)	
Never had surgery	279 (39.9)
Surgery without PONV	377 (53.9)
Surgery with PONV	43 (6.2)
History of motion sickness, no. (%)	154 (22.0)
Mean hemoglobin ± SD, g/l	123 ± 18
Mean hematocrit ± SD, %	37 ± 5
Mean creatinine ± SD, μmol/l	59 ± 13
Intraoperative intervention data, mean ± SD	
Duration of anesthesia, min	175 ± 74
Propofol, mg	958 ± 437
Remifentanil, mg	1.2 ± 0.7
Sufentanil, mcg	32 ± 15
Crystalloid, ml	1512 ± 575
Estimated blood loss, ml	69 ± 93
Urine output, ml	369 ± 261
Intraoperative monitoring data, mean ± SD^a^	
Mean respiratory rate, breath per min	14 ± 2
Mean end-tidal carbon dioxide, mmHg	34 ± 4
Mean heart rate, beat per min	66 ± 9
Mean systolic blood pressure, mmHg	117 ± 12
Mean diastolic blood pressure, mmHg	73 ± 8
Mean MAP, mmHg	86 ± 9
Mean pulse oxygen saturation, %	100 ± 1
Mean body temperature, °C	36 ± 1
Mean muscular tissue oxygen saturation, %	83 ± 7
Postoperative QoR	
QoR-15 score, mean ± SD	121 ± 19
QoR-15 score, median [IQR]	122 [109-135]
Number of patients with a QoR-15 ≥122, no. (%)	354 (50.6)

*SD* standard deviation, *ASA* American Society of Anesthesiologists, *PONV* postoperative nausea and vomiting, *MAP* mean arterial pressure, *QoR* quality of recovery, *IQR* interquartile range

^a^For time-series data, we first removed those outliers defined as the data outside of the 0.5th–99.5th percentile. The mean of all data within the 0.5th–99.5th percentile was first derived for each patient. These means were then averaged to derive the mean for all patients

### Model discrimination

Models’ performance is presented in Table 3. When using the preoperative data only, all four models exhibited poor performance, with AUROCs ranging from 0.65 to 0.68. The inclusion of the intraoperative intervention and/or monitoring data improved the performance of the deep leaning, logistic regression, and random forest models, but not the support vector machine model, which had an AUROC that remained in the range of 0.65–0.71.

**Table 3:** Models’ performance based on different datasets

	Accuracy^a^	Sensitivity^a^	Specificity^a^	F1 score^a^	AUROC
Preoperative data					
Deep learning	0.61 (0.57–0.65)	0.61 (0.54–0.68)	0.61 (0.50–0.71)	0.60 (0.56–0.64)	0.65 (0.62–0.67)
Logistic regression	0.63 (0.59–0.66)	0.62 (0.59–0.65)	0.63 (0.56–0.71)	0.62 (0.60–0.65)	0.68 (0.66–0.70)
Support vector machine	0.61 (0.56–0.66)	0.51 (0.40–0.62)	0.70 (0.59–0.81)	0.56 (0.49–0.63)	0.65 (0.60–0.70)
Random forest	0.62 (0.60–0.65)	0.59 (0.49–0.70)	0.66 (0.59–0.72)	0.60 (0.55–0.66)	0.68 (0.65–0.70)
Intraoperative intervention data					
Deep learning	0.74 (0.70–0.79)	0.73 (0.66–0.80)	0.74 (0.61–0.87)	0.74 (0.71–0.77)	0.79 (0.75–0.82)
Logistic regression	0.76 (0.71–0.81)	0.77 (0.73–0.80)	0.76 (0.64–0.88)	0.76 (0.73–0.79)	0.78 (0.74–0.82)
Support vector machine	0.59 (0.54–0.64)	0.50 (0.41–0.59)	0.67 (0.55–0.80)	0.54 (0.48–0.60)	0.65 (0.61–0.68)
Random forest	0.73 (0.67–0.79)	0.75 (0.71–0.78)	0.72 (0.58–0.86)	0.74 (0.69–0.78)	0.81 (0.76–0.85)
Intraoperative monitoring data					
Deep learning^b^	0.70 (0.69–0.72)	0.64 (0.58–0.69)	0.77 (0.70–0.84)	0.68 (0.66–0.71)	0.77 (0.72–0.81)
Logistic regression^c^	0.69 (0.63–0.75)	0.68 (0.64–0.72)	0.69 (0.56–0.83)	0.68 (0.64–0.73)	0.72 (0.68–0.77)
Support vector machine^c^	0.62 (0.58–0.66)	0.61 (0.56–0.66)	0.63 (0.52–0.75)	0.62 (0.59–0.64)	0.68 (0.65–0.71)
Random forest^c^	0.61 (0.57–0.65)	0.83 (0.72–0.94)	0.40 (0.21–0.58)	0.68 (0.66–0.69)	0.74 (0.73–0.76)
Intraoperative monitoring data + SmtO_2_					
Deep learning^b^	0.71 (0.69–0.73)	0.64 (0.57–0.72)	0.77 (0.68–0.87)	0.69 (0.67–0.70)	0.77 (0.74–0.79)
Logistic regression^c^	0.69 (0.63–0.75)	0.68 (0.64–0.72)	0.69 (0.54–0.85)	0.69 (0.65–0.72)	0.73 (0.68–0.78)
Support vector machine^c^	0.67 (0.64–0.70)	0.63 (0.57–0.69)	0.70 (0.61–0.79)	0.65 (0.63–0.68)	0.71 (0.67–0.76)
Random forest^c^	0.65 (0.60–0.70)	0.87 (0.79–0.95)	0.44 (0.27–0.61)	0.71 (0.70–0.73)	0.78 (0.73–0.82)
Preoperative data + intraoperative monitoring data + intraoperative intervention data					
Deep learning	0.73 (0.70–0.76)	0.74 (0.69–0.80)	0.71 (0.62–0.80)	0.73 (0.71–0.75)	0.81 (0.78–0.83)
Logistic regression	0.73 (0.66–0.80)	0.75 (0.70–0.80)	0.72 (0.58–0.85)	0.74 (0.68–0.79)	0.77 (0.70–0.85)
Support vector machine	0.59 (0.56–0.61)	0.50 (0.40–0.60)	0.67 (0.56–0.78)	0.54 (0.49–0.59)	0.65 (0.61–0.69)
Random forest	0.76 (0.72–0.80)	0.82 (0.75-0.88)	0.70 (0.57–0.83)	0.77 (0.74–0.80)	0.82 (0.78–0.87)

Data are presented as mean (95% confidence interval)

*AUROC* area under the receiver operating characteristic curve, *SmtO*_*2*_ muscular tissue oxygen saturation

^a^Calculated based on the decision threshold of 0.5

^b^Based on time-series data

^c^Based on the maximum, minimum, mean, and standard deviation values of time-series data

In this study, performance was defined as indistinct if the AUROC’s 95% confidence interval (CI) overlaps. The deep learning model had indistinct performance when using the intraoperative intervention data only (AUROC, 0.79; 95% CI, 0.75–0.82), using the intraoperative monitoring data only (AUROC, 0.77; 95% CI, 0.72–0.81), and using the preoperative, intraoperative intervention, and monitoring data combined (AUROC, 0.81; 95% CI, 0.78–0.83).

The logistic regression model had indistinct performance when using the intraoperative intervention data only (AUROC, 0.78; 95% CI, 0.74–0.82), using the intraoperative monitoring data only (AUROC, 0.72; 95% CI, 0.68–0.77), and using the preoperative, intraoperative intervention, and monitoring data combined (AUROC, 0.77; 95% CI, 0.70–0.85).

The random forest model had indistinct performance when using the intraoperative intervention data only (AUROC, 0.81; 95% CI, 0.76–0.85) and using the preoperative, intraoperative intervention and monitoring data combined (AUROC, 0.82; 95% CI, 0.78–0.87). In contrast, the performance was inferior when using the intraoperative monitoring data only (AUROC, 0.74; 95% CI, 0.73–0.76).

### Model calibration

**Fig. 4:**
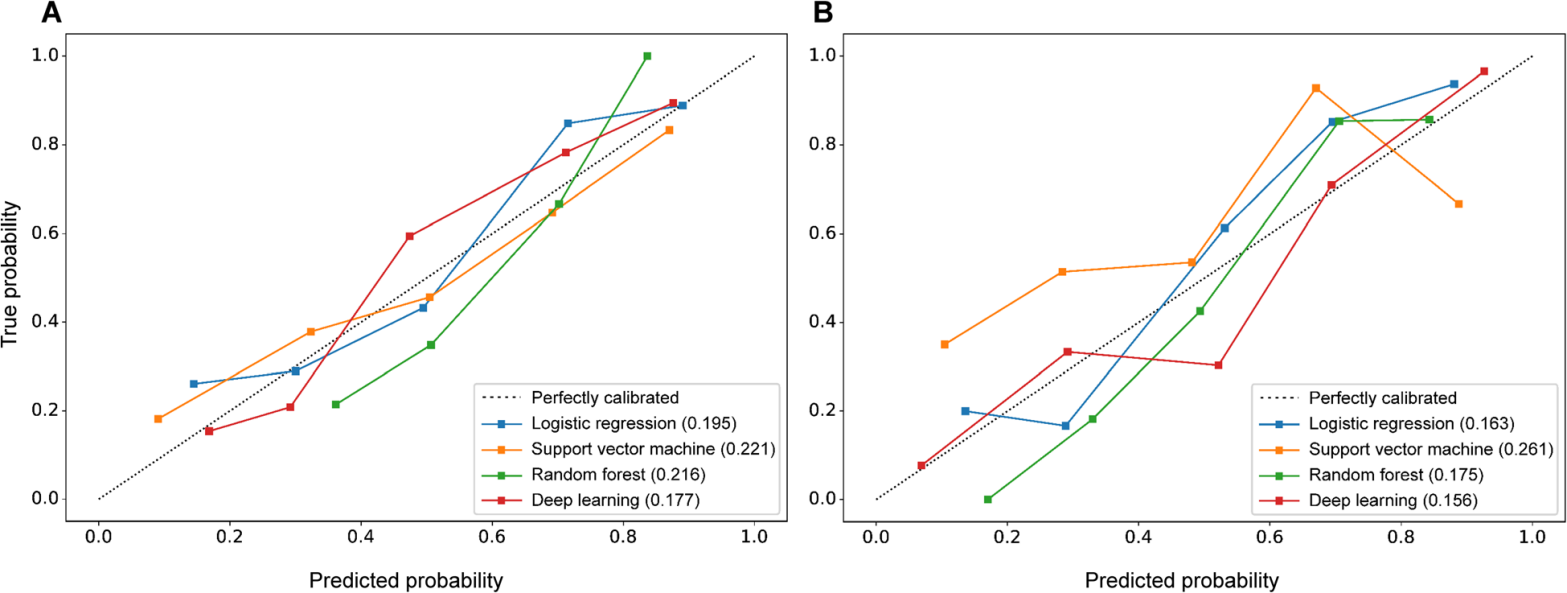
Calibration plots and Brier scores of different models. Models based on intraoperative monitoring data (**a**) and based on preoperative data, intraoperative monitoring data, and intraoperative intervention data (**b**) are presented

The calibration plots and Brier scores are shown in Fig. 4. Compared to the logistic regression, support vector machine, and random forest models, the deep learning model exhibited better calibration when using the intraoperative monitoring data only (Brier score=0.177, Fig. 4a) and when using the preoperative, intraoperative intervention, and monitoring data combined (Brier score=0.156, Fig. 4b).

### Feature importance

**Fig. 5:**
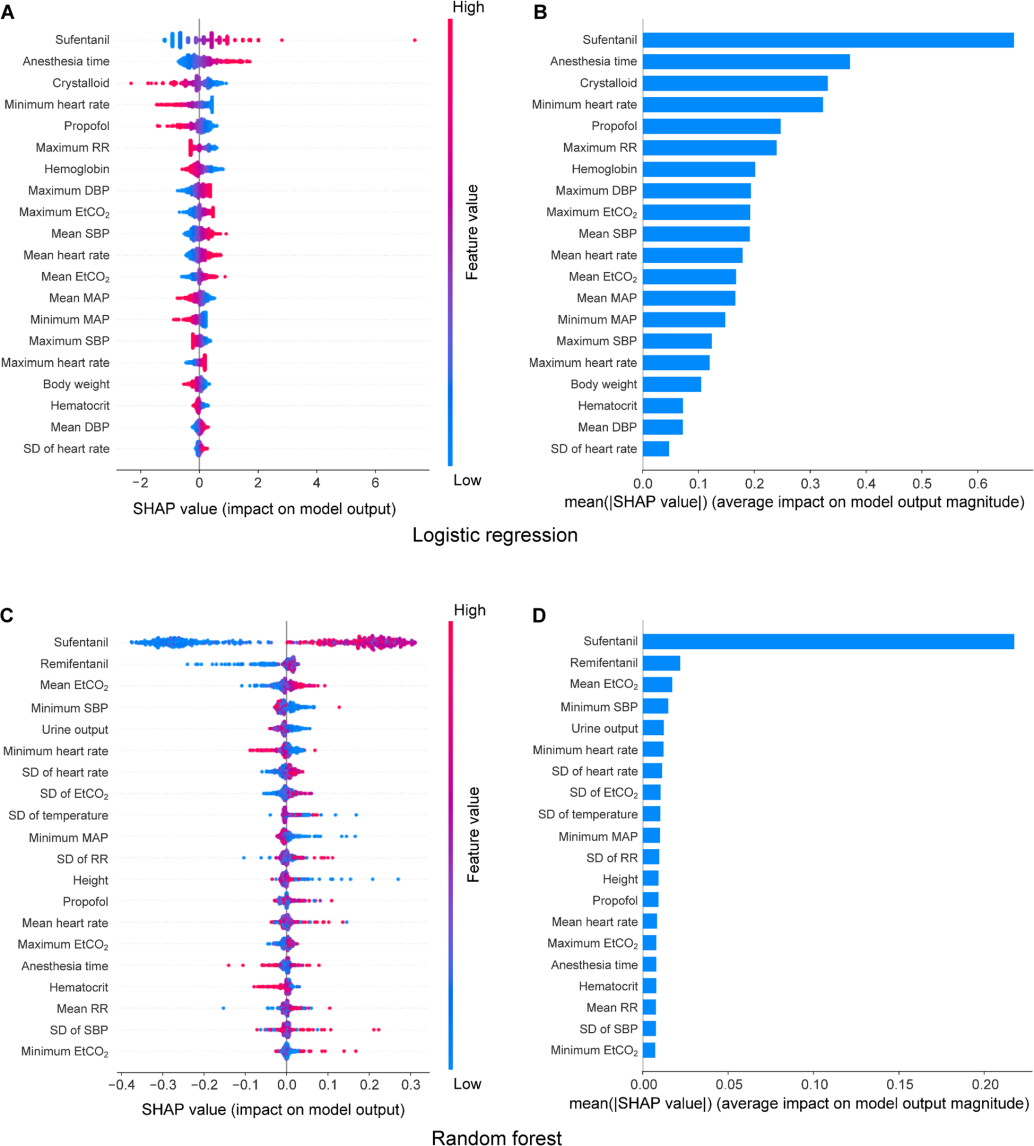
SHAP summary plot and feature ranking. SHAP values for the twenty most important features used in the logistic regression model (**a**, **b**) and random forest model (**c**, **d**) are shown. In plots **a** and **c**, each point represents a specific feature’s SHAP value in an individual patient. In plots **b** and **d**, a specific feature’s absolute SHAP values for all patients were averaged. The larger a feature’s absolute SHAP value, the larger the impact of the feature on patient’s outcome. A positive and negative SHAP value corresponds to a higher and lower likelihood of having an unsatisfactory outcome, respectively. The mean absolute SHAP value of all patients reflects the significance of the feature in driving model’s prediction, i.e., the higher the mean, the more significant the feature for prediction and vice versa. In plots **a** and **c**, the actual value of the feature for each patient is color-coded, with red color representing higher values and blue color representing lower values. Of note, a specific feature’s SHAP value and actual value are different. SHAP SHapley Additive exPlanation, RR respiratory rate, DBP diastolic blood pressure, EtCO_2_ end-tidal carbon dioxide, SBP systolic blood pressure, MAP mean arterial pressure, SD standard deviation

SHAP values for the logistic regression and random forest models are presented in Fig. 5. We did not present SHAP values for the deep leaning and support vector machine models due to the unsuitability of using the SHAP method to explain the InceptionTime deep learning model and the poor performance of the support vector machine model in our study. Among features utilized in modeling, the dose of sufentanil administered during surgery appeared to have the most significant contribution to the prediction of the post-hysterectomy quality of recovery (Fig. 5b, d). A higher dose of sufentanil was associated with a higher likelihood of having an unsatisfactory quality of recovery (Fig. 5a, c).

### Class activation mapping

Examples of class activation mapping are presented in eFigure 1 in Additional file 1. Overall, no specific parts of the temporal input appeared to have peculiar contributions.

## Discussion

### Summary of findings

We performed the first study investigating the prognostication of the quality of recovery using a deep learning model based on intraoperative time-series monitoring data in surgical patients. Our study has some unique findings. First, we found that the deep learning model based only on the intraoperative time-series monitoring data was able to predict the quality of recovery after laparoscopic hysterectomy. When using intraoperative monitoring data only, the performance of the deep learning model was better than the logistic regression and random forest models. This finding attests to the potential value of using the intraoperative time-series monitoring data for outcome prediction. Second, we found that inclusion of the intraoperative intervention and/or monitoring data significantly improved the performance of the deep learning, logistic regression, and random forest models compared to inclusion of the preoperative data only. This finding suggests that the performance of these models is input data-dependent. It also substantiates the close relationship between intraoperative management and postoperative outcomes. Third, we found that use of the preoperative, intraoperative intervention, and monitoring data combined did not significantly improve the models’ performance compared to the use of intraoperative intervention data only or the use of the intraoperative monitoring data only. This finding suggests certain inherent associations among different datasets.

### Comparison with the current literature

Machine learning recently began to make its footprint in the field of perioperative medicine (Hashimoto, Witkowski, Gao, Meireles, & Rosman, 2020). Models based on deep learning algorithms have been developed for postoperative mortality prediction. Lee et al. developed a deep neural network model based on data from 59,985 patients to predict postoperative in-hospital mortality (Lee, Hofer, Gabel, Baldi, & Cannesson, 2018). The AUROC of Lee et al.’s model was 0.88 (Lee et al., 2018). Fritz et al. developed a multipath convolutional neural network model based on data from 95,907 patients to predict 30-day postoperative mortality with an AUROC of 0.87 (Fritz et al., 2019).

Our study distinguished itself from these previous studies in the following aspects: (1) we targeted the quality of recovery in a relatively homogenous, young, and healthy female surgical patient population, while previous work targeted mortality in heterogeneous patient populations; (2) we investigated the models’ performance based on different types of input data, while previous work did not; (3) our models were based on prospectively collected data, while previous work was based on retrospective data; (4) we used InceptionTime (Fawaz et al., 2019a, b), a state-of-the-art deep learning model, in our study, while previous work used different algorithms; and (5) we used high-frequency time-series monitoring data collected throughout the entire surgery, while Lee et al. did not use time-series data, and Fritz et al. used only time-series data collected over a random 60-min interval.

### Limitations

Our study has limitations. First, our study was performed in relatively young and healthy female patients; therefore, caution is needed when generalizing our models to other patient populations (Moons et al., 2019). Second, the models’ performances in our study were lower than that in Lee et al.’s study (Lee et al., 2018) and Fritz et al.’s study (Fritz et al., 2019); among the different potential causes for this inferiority, the most likely cause is the small sample size of our study. The model’s accuracy becomes less when the sample size becomes smaller (Wisz et al., 2008). Third, it may seem arbitrary when we used a cutoff QoR-15 value of 122 to dichotomize the quality of recovery. However, this value was also adopted by a previous study (Kleif & Gögenur, 2018) and is consistent with the mean and median QoR-15 scores of our patients (Li et al., 2020). Fourth, our models did not use some intraoperative data, e.g., the type and dose of vasoactive drugs. However, the vasoactive drugs may exert their characteristic footprints in time-series monitoring data. Thus, the use of time-series monitoring data might have made up for the omission of certain intervention information in modeling. Fifth, we did not time stamp the intraoperative intervention data; instead, we used the total at the end of surgery in modeling. This approach might have ignored certain information that is valuable for modeling.

## Conclusions

Deep learning based on the intraoperative time-series monitoring data can predict the quality of recovery after laparoscopic hysterectomy. The performance of the deep learning, logistic regression, and random forest models is input data-dependent. The inclusion of the intraoperative intervention and/or monitoring data significantly improved the models’ performance compared to the inclusion of preoperative data only. These models may help clinicians identify at-risk patients, adjust perioperative care, and continuously improve the quality of clinical care. Our study should be regarded as a preliminary step towards accomplishing machine learning prediction based on intraoperative time-series monitoring data due to the various limitations discussed above. Moving forward, our models need to be validated using large-scale datasets with different patient populations.

## Supplementary Information

**Additional file 1: eTable 1.** Performance of the deep learning model based on intraoperative monitoring data per different scaling methods. **eTable 2.** The maximum and minimum values used for continuous variable scaling. **eFigure 1.** Class activation mapping in one patient. The degree of the contribution to prognostication is color coded, with red corresponding to a higher contribution and blue to a lower contribution. A. Deep learning model based on intraoperative monitoring data. B. Deep learning model based on preoperative data + intraoperative intervention data + intraoperative monitoring data

## Data Availability

Please contact Lingzhong Meng (lingzhong.meng@yale.edu).
