# Supplementary material for: Can a deep learning model based on intraoperative time-series monitoring data predict post-hysterectomy quality of recovery?
Source: Perioper Med (Lond). 2021 Apr 6;10:8. doi: 10.1186/s13741-021-00178-4 (PMC8022389; doi:10.1186/s13741-021-00178-4)

**Additional File 1**

Supplement to: **Can a deep learning model based on intraoperative time-series monitoring data predict post-hysterectomy quality of recovery?**

**eTable 1.** Performance of the deep learning model based on intraoperative monitoring data per different scaling methods

|  | Accuracy | Sensitivity | Specificity | F1 score | AUROC |
| --- | --- | --- | --- | --- | --- |
| Raw data* | 0.73 (0.67-0.80) | 0.63 (0.52-0.73) | 0.84 (0.75-0.93) | 0.70 (0.62-0.77) | 0.79 (0.72-0.86) |
| 1000 time points† | 0.70 (0.69-0.72) | 0.64 (0.58-0.69) | 0.77 (0.70-0.84) | 0.68 (0.66-0.71) | 0.77 (0.72-0.81) |
| 4000 time points† | 0.71 (0.71-0.71) | 0.63 (0.60-0.67) | 0.79 (0.76-0.82) | 0.69 (0.67-0.70) | 0.76 (0.73-0.78) |

Data are presented as mean (95% confidence interval).

* The time-series data were not scaled before entering the deep learning model.

† The time-series data were scaled to the same length of 1,000/4,000 time points using the standard down-sampling or up-sampling (spline interpolation) methods before entering the deep learning model.

AUROC, area under the receiver operating characteristics curve.

**eTable 2.** The maximum and minimum values used for continuous variable scaling

| Variable | Maximum | Minimum |
| --- | --- | --- |
| Age, year | 65 | 27 |
| Height, cm | 177 | 145 |
| Body weight, kg | 100 | 33 |
| Body mass index, kg/m^2^ | 38 | 14 |
| Hemoglobin, g/l | 165 | 65 |
| Hematocrit, % | 49 | 22 |
| Creatinine, μmol/l | 216.75 | 31 |
| Sufentanil, mcg | 170 | 0 |
| Remifentanil, mg | 5.6 | 0.1 |
| Propofol, mg | 2810 | 160 |
| Anesthesia time, min | 434 | 43 |
| Crystalloid, ml | 4500 | 300 |
| Blood loss, ml | 1200 | 5 |
| Urine output, ml | 1700 | 20 |
| Body temperature, °C | 38 | 33 |
| Respiratory rate, breath per min | 34 | 10 |
| End-tidal carbon dioxide, mmHg | 54.5 | 21 |
| Systolic blood pressure, mmHg | 174 | 80 |
| Diastolic blood pressure, mmHg | 108 | 44 |
| Mean arterial pressure, mmHg | 127 | 55 |
| Pulse rate, beat per min | 108 | 44 |
| Pulse oxygen saturation, % | 100 | 94 |
| Muscular tissue oxygen saturation, % | 95 | 52 |

**eFigure 1.** Class activation mapping in one patient. The degree of the contribution to prognostication is color coded, with red corresponding to a higher contribution and blue to a lower contribution.

1. Deep learning model based on intraoperative monitoring data


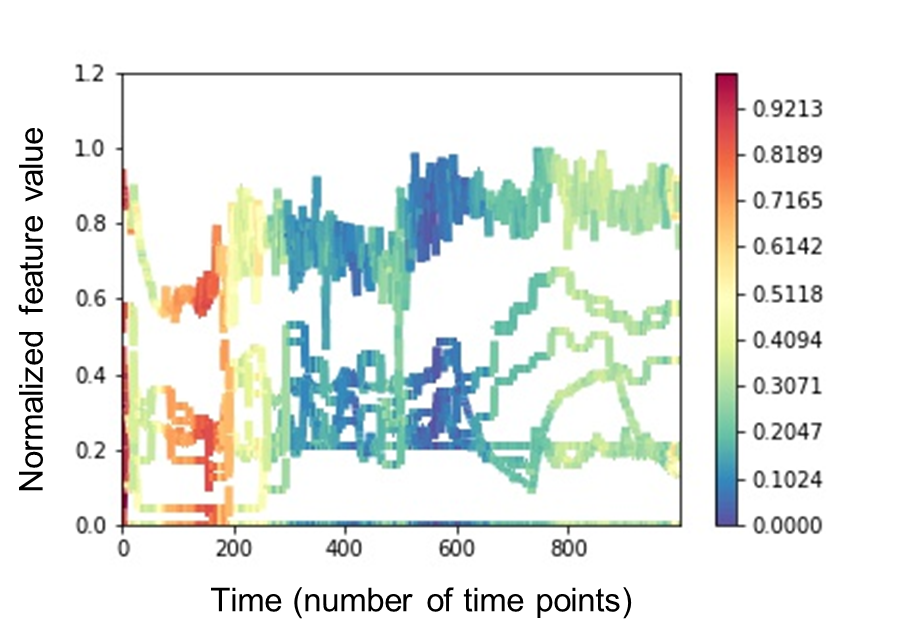


1. Deep learning model based on preoperative data + intraoperative intervention data + intraoperative monitoring data


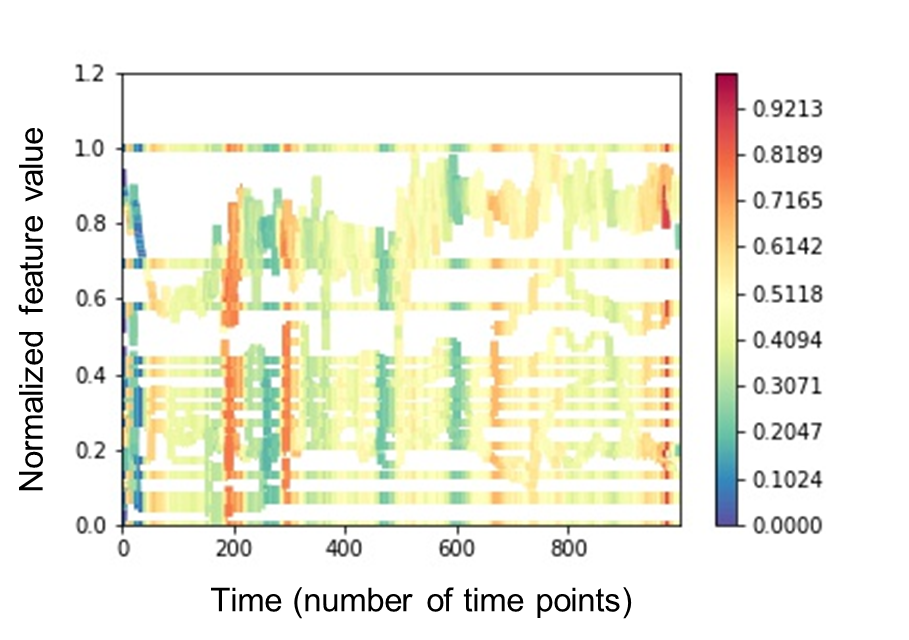

Supplement: Supplementary file 1 — Additional file 1: eTable 1. Performance of the deep learning model based on intraoperative monitoring data per different scaling methods. eTable 2. The maximum and minimum values used for continuous variable scaling. eFigure 1. Class activation mapping in one patient. The degree of the contribution to prognostication is color coded, with red corresponding to a higher contribution and blue to a lower contribution. A. Deep learning model based on intraoperative monitoring data. B. Deep learning model based on preoperative data + intraoperative intervention data + intraoperative monitoring data [file 13741_2021_178_MOESM1_ESM.docx]
